# Modeling gradually changing seasonal variation in count data using state space models: a cohort study of hospitalization rates of stroke in atrial fibrillation patients in Denmark from 1977 to 2011

**DOI:** 10.1186/1471-2288-12-174

**Published:** 2012-11-20

**Authors:** Anette L Christensen, Søren Lundbye-Christensen, Kim Overvad, Lars H Rasmussen, Claus Dethlefsen

**Affiliations:** 1 Department of Cardiology, Aalborg AF Study Group, Center for Cardiovascular Research, Aalborg Hospital, Aarhus University Hospital, Aalborg, Denmark; 2 Department of Epidemiology, School of Public Health, Aarhus University, Aarhus, Denmark; 3 Thrombosis Research Unit, Faculty of Medicine, Aalborg University, Aalborg, Denmark

## Abstract

**Background:**

Seasonal variation in the occurrence of cardiovascular diseases has been recognized for decades. In particular, incidence rates of hospitalization with atrial fibrillation (AF) and stroke have shown to exhibit a seasonal variation. Stroke in AF patients is common and often severe. Obtaining a description of a possible seasonal variation in the occurrence of stroke in AF patients is crucial in clarifying risk factors for developing stroke and initiating prophylaxis treatment.

**Methods:**

Using a dynamic generalized linear model we were able to model gradually changing seasonal variation in hospitalization rates of stroke in AF patients from 1977 to 2011. The study population consisted of all Danes registered with a diagnosis of AF comprising 270,017 subjects. During follow-up, 39,632 subjects were hospitalized with stroke. Incidence rates of stroke in AF patients were analyzed assuming the seasonal variation being a sum of two sinusoids and a local linear trend.

**Results:**

The results showed that the peak-to-trough ratio decreased from 1.25 to 1.16 during the study period, and that the times of year for peak and trough changed slightly.

**Conclusion:**

The present study indicates that using dynamic generalized linear models provides a flexible modeling approach for studying changes in seasonal variation of stroke in AF patients and yields plausible results.

## Background

Epidemiological studies on seasonal variation i.e. cyclic behavior within a given time period, have been published for several decades. A variety of statistical methods to describe seasonal variations have been employed. These methods range from *χ*^2^testing of difference in frequencies to linear regression. In particular, the model derived by Edwards in 1961 [1] has been employed in epidemiological studies of seasonal variation in frequencies of the occurrence of diseases [2-5]. In recent years, the Poisson regression model has also been employed as an alternative to Edwards’ model [6-9]. This method facilitates adjustments for possible confounders and straightforward modeling of effect modifiers, as opposed to Edwards’ model [10].

Data investigated for seasonal variation are often referred to as time series. Time dependency between data is an essential characteristic of time series; hence both Edwards’ model and Poisson regression may provide non-valid conclusions, due to violation of the model assumption of independent data. Furthermore, underlying risk factors may change over time, affecting the seasonal variation, and it is therefore desirable to be able to model gradual changes in covariates over time in time series [11,12]. This is featured when applying the dynamic generalized linear models (DGLMs) [13], a generalization of the generalized linear models (GLMs) introduced by Nelder and Wedderburn, 1972 [14]. These models enable modeling of time series with distributions belonging to the natural exponential family, e.g. Poisson, binomial, and negative binomial distributions. Specification of the mean value structure and interpretation of estimated coefficients are similar to ordinary regression analysis. Other models used to describe seasonal variation accounting for the dependency between observations have been proposed [15-19].

In the paper by Lundbye-Christensen et al., 2009, a similar approach for modeling seasonal variation of discharge data on acute myocardial infarction in Denmark has been employed [11]. The authors provide an example of the use of DGLMs by modeling an overall trend and seasonal variation present in data and explain the interpretation of model coefficients and results. However, the correlation structure and estimation of unknown hyperparameters are not described in detail despite the essentials of this issue.

The aim of this study was to illustrate how to model gradually changing seasonal variation using a DGLM and to estimate unknown hyperparameters in the context of epidemiology by analyzing data consisting of weekly frequencies of hospitalizations with stroke in AF patients in the Danish population from 1977 to 2011. We outline a specific mean value structure adapted to model seasonal variation of hospitalizations with stroke in patients with AF and propose an algorithm to estimate variance parameters and outline estimation of regression estimates and calculation of model diagnostics. Furthermore, a free available implementation of the approach is developed and applied to data.

## Methods

### Study population

The study population consisted of all residents in Denmark who were hospitalized in a Danish hospital with a diagnosis of AF from 1977 to 2011 and was retrieved from the Danish National Patient Registry. This registry holds information on hospitalizations in public hospitals in Denmark. Each record includes information on date of admission and discharge, primary and secondary diagnoses, hospital department, and identification of the patient in terms of the Danish civil registry number, which is a unique identification number given to all Danish residents at birth. For further information we refer to [20]. Diagnoses were registered according to the International Classification of Diseases (ICD), version 8 from 1977 to 1994 and version 10 from 1994 to 2011. Information on vital status and emigration was retrieved from the Danish Civil Registration System. For further information on this registry we refer to [21].

Diagnoses of AF were identified according to ICD-8 codes 427.93 and 427.94 and ICD-10 code I48. The study population was followed until a possible hospitalization with a diagnosis of stroke (ICD-8: 433, 434, and 436, ICD-10: I63 and I64), death, emigration, or end of follow-up, which was set at August 1, 2011, and date of possible event was registered. Stroke in AF patients was defined as a first-time hospitalization with stroke for patients having at least one previous hospitalization with AF. Hospitalizations with both stroke and AF diagnoses were also considered as hospitalizations with stroke in AF patients. To reduce inclusion of prevalent cases of AF and stroke in AF patients, identified cases before 1980 were excluded from the analysis.

### Statistical methods

The objective of the statistical analysis is to model changing seasonal variation. Using ordinary Poisson regression, we may analyze consecutive time periods simultaneously, e.g. divide the study period into decades. However, several problems occur when doing so. First, the change between time periods is abrupt, which may seem as a crude assumption. Second, only data within a given time period are considered when estimating the seasonal variation. Letting the time periods become shorter and shorter, the changes from time periods may be less abrupt, and the amount of data becomes smaller providing larger uncertainty on estimates. Nevertheless, the model assumption of independent data is violated, causing potentially non-valid results.

Considering the DGLM from an intuitive perspective, when analyzing weekly data, the estimation of the seasonal variation is performed for every week by considering the nearest 52 weeks and estimating the seasonal variation based on these data. The constraints imposed on the model ensure that the estimated seasonal variation only changes slightly when moving the 52-week window one week forward.

A DGLM consists of three parts. First, the multidimensional state model which specifies how the underlying unobserved process is generated. Second, the observation model which specifies the relation between the latent process and the data, the latter being considered as indirect measurements of the state model, and finally, the initial state model which specifies the initialization of the latent process. The dynamic nature of DGLMs originates in the first-order Markovian evolution of the regression coefficients, which means that the coefficients are allowed to change over time. Modeling gradually changing seasonal variation in incidence rates of hospitalizations with stroke in AF patients, we assumed that both the state model and the initial state model were Gaussian distributed and the observation model was Poisson distributed.

Denoting the time series of frequencies of hospitalizations with stroke in AF patients by {*y*_*t*_|*t *= 1,…,*n*} and corresponding number of subjects at risk by offset_*t*_, the DGLM is formally denoted

$$\begin{array}{l} \;y_{t}|\eta_{t}∼\text{Poisson}({\text{offset}}_{t}\lambda_{t})\\ \;\eta_{t}=\log(\lambda_{t})=F_{t}^{⊤}\theta_{t}\\ \;\theta_{t}=G_{t}\theta_{t-1}+\omega_{t},\;\omega_{t}∼N[0,W]\\ \;\;\theta_{0}∼N[m_{0},C_{0}] \end{array}$$

where *ω*_*t*_’s are serially uncorrelated and zero-mean Gaussian distributed with unknown and constant covariance matrix *W*. The latent process is denoted by *θ *= (*θ*_1_,…,*θ*_*n*_) and is initialized by the initial state model. It is assumed that the matrices *F*_*t*_ and *G*_*t*_ are known along with *m*_0 _and *C*_0_. However, all matrices may depend on a set of hyperparameters, denoted by the vector *φ*.

In case of both state model and observation model being Gaussian, inference on the latent process is obtained by use of the Kalman filter which results in conditional densities. The filter is a recursive updating scheme that updates the DGLM whenever a new data point is available. The Kalman smoother is a backward recursive updating scheme that updates the state model conditional on all data. Hence, Kalman smoothing is a post estimation procedure.

When dealing with non-Gaussian observation models, the Kalman filter and smoother are not immediately applicable. Given the above model, the derivation of the Kalman filter breaks down due to the non-normality of the observation model. As proposed by Durbin and Koopman, 2001 [22], the DGLM is linearized by calculating the first two derivatives of the observation model in a given trial value of the state vector and identifying an approximating Gaussian observation model. This is performed iteratively by applying the Kalman filter and smoother on the approximating Gaussian model to obtain a new trial value of the state vector until convergence is reached. Upon convergence, the likelihood function of the approximating Gaussian model has the same mode and curvature at the mode as the original DGLM. This procedure is commonly referred to as iterated extended Kalman smoothing. All inference procedures assume all matrices being known, including the covariance matrix *W*.

The matrices *F*_*t *_and *G*_*t*_ operate as design matrices for the observation model and the state model and are commonly specified by the modeler for all *t*. In contrast, the covariance matrix *W* which specifies the correlation structure both over time and between coordinates in the evolution of *θ*_*t*_, may be unknown and has to be estimated. Given that this covariance matrix is parameterized by some hyperparameters, the objective is to estimate these. Estimation of hyperparameters in a DGLM has been recommended to be performed using the EM algorithm [23] initially followed by an iterative numerical optimization algorithm [24-26].

The EM algorithm is a two-step numerical iterative estimation procedure based on maximization of the likelihood function, with the characteristic that for each step the likelihood function does not decrease and that convergence to a stationary point for DGLMs is obtained [24]. We applied the EM algorithm initially to estimate the hyperparameters in order to reach the region of maximum of the likelihood function [26]. A convergence criterion, in terms of either a maximum number of iterations or a given threshold, has to be provided by the modeler and may depend on the context.

Upon convergence of the EM algorithm, the estimation procedure is switched to another iterative numerical optimization method in order to pinpoint the actual maximum by maximizing the log likelihood function, since convergence of the EM algorithm may be rather slow [26]. However, the log likelihood function of a DGLM is not obtainable on closed form; hence, estimation based on sampling may be utilized. As proposed by Durbin and Koopman, 2001 [22], we calculated the exact log likelihood value by use of importance sampling. This sampling technique is a variance reduction simulation scheme which ensures efficient computation time. For further description we refer to [22].

For the purpose of this study, we specified the linear predictor, *η*_*t*_, as a sum of two terms: a secular trend, *T*_*t*_ and a seasonal variation, *S*_*t*_. The secular trend was modeled as a local linear trend which, in the context of DGLM, is not restricted to only increase or decrease constantly. In fact, the trend is allowed to change in both directions due to the dynamic nature of the model. The seasonal variation is modeled as a sum of two sinusoids with periods of 12 and 6 months, respectively. This specification allows for some asymmetry of the seasonal variation. The choice of the numbers of sinusoids to describe the seasonal variation is a trade off between having a flexible model and reducing the complexity of the model, i.e. keeping the number of parameters low. We aggregated the data to seven-day intervals in order to eliminate a possible week-day-effect and analyzed data from 1980 to 2011. The person-time at risk was used as offset, and incidence rates per 100 person-years of stroke in AF patients were analyzed, The peak-to-trough (PTT) ratios were calculated. This PTT ratio is often reported as a measurement of the intensity of seasonal variation in incidence rates and is an incidence rate ratio. Adjustment for possible confounding factors and modeling effect modifiers may be performed with these models, but is, however, beyond the scope of this paper, as we outline the basic usage of the DGLMs in modeling seasonal variation.

Imposing a specific structure on the evolution covariance matrix, *W*, we may ensure separation and interpretation of the coefficients in the state vector [27]. This structure needs contextual considerations and may not be trivial to determine. For this study, we assumed that the two terms, the trend and the seasonal variation, were independent, and hence, *W* became a block diagonal matrix. Imposing only strictly positive variances, all parameters of the model were allowed to change over time. No structure was assumed for the first block matrix corresponding to the covariance matrix of the trend, for which reason the variance of the level was determined without regard to the variance of the slope, and, in addition, the level and slope were allowed to be dependent, meaning that the covariance was non-zero. We denote the variance of the level by *φ*_1_, and the variance of the slope by *φ*_2_. The covariance of *φ*_1 _and *φ*_2_ is denoted by *φ*_3_.

Contrarily, we assumed a specific structure for the second block matrix, corresponding to the covariance matrix of the seasonal variation. Acknowledging that the cosine function is merely an appropriate phase shift of the sine function, the two coefficients of each sinusoid have equal variances but are allowed to differ between the two sinusoids. The variance of the first sinusoid is denoted by *φ*_4_, and the variance of the second is denoted by *φ*_5_. Furthermore, the two coefficients of each sinusoid were dependent, hence non-zero covariances, denoted by *φ*_6_ for the first sinusoid and *φ*_7 _for the second. Consequently, the second block matrix consists of two pairwise equal variances in the diagonal and two covariances. Furthermore, the four covariances between the four coefficients of the two sinusoids were assumed equal, denoted by *φ*_8_.

These constraints provided a covariance matrix, *W*, which was parameterized by an eight-dimensional hyperparameter vector, consisting of four variances and four covariances. The specification of the covariance matrix ensures that possible overdispersion in the count data is accounted for, since an additional variance term, besides the normal Poisson variance, is present. Also, serial correlation in data is modeled by allowing non-zero variances in *W*[28].

In order to assess whether the seasonal variation was changing over time, we also fitted a DGLM with the only difference being that the covariance matrix of the seasonal variation contained only zero entries, corresponding to a static seasonal variation. Akaike’s Information Criterion (AIC) was calculated for both models, and the model with lowest AIC was considered parsimonious and in favor of the data [29].

To identify possible misspecifications of this model, we performed a residual analysis based on residuals proposed by Jørgensen et al., 1999 [28]. The authors define several types of residuals for both the observation model and the latent model, based on either the Kalman filter or smoother, each of which may reveal misspecification of the corresponding model. We considered the residuals of the approximating Gaussian model provided by iterated extended Kalman smoothing.

Statistical software to perform the above calculation is freely available using R[30]. Specification of the DGLM is easily performed using the package sspir, which also includes an implementation of the EM algorithm, specific to this kind of models, Kalman filtering and smoothing, and iterated extended Kalman smoothing [31,32]. Using the package KFAS, calculation of the exact likelihood function is available [33] and, along with standard non-linear optimization algorithm implemented in R, the hyperparameters may be estimated. This procedure is implemented in the function rrd in the package Peak2Trough. For further information we refer to [34]. All analyses were performed using R version 2.13.2 on a 64bit Intel®Core 2 E7300 2.66GHz CPU 4GB RAM with Ubuntu 11.10.

## Results

We identified 270,017 first-time hospitalizations with AF (48% females) from January 1, 1980 to August 1, 2011, corresponding to 1,648 weeks. In total, 507 subjects were lost to follow-up due to emigration or registered as missing persons, whereas 145,378 subjects died before end of follow-up.

The median age at AF diagnosis was 79 years for females, and 72 years for males. During a median follow-up time of 2.8 years, 39,632 subjects were hospitalized with a first-time stroke (55% females). Median time to stroke was 348 days, and one third was diagnosed with both AF and stroke during the same hospitalization. The median weekly frequency of AF hospitalizations was 153, and 23 for stroke in AF patients.

We initialized the DGLM according to a multivariate Gaussian distribution with zero-mean vector and covariance matrix, *C*_0 _= 100*I*_6_. For the purpose of estimating the unknown covariance matrix, *W *, the initial value of the hyperparameter vector was *φ*_0 _= 10^−4^(10,0*.*01,0*.*001,3,3,1,1,1).

Convergence for the EM algorithm was chosen to be when either the difference in log likelihood function value between two steps was smaller than 0.1 or after a maximum of 1,000 iterations. The EM algorithm converged after 334 iterations. The computation time was 16 minutes. Switching to a numerical non-linear optimization algorithm in terms of the nlm function in R, we obtained a maximum log likelihood value for the DGLM of 98.7 after 3 minutes for hyperparameter values equal to $\hat{\varphi }=10^{-7}(410,0.003,0.01,8,0.05,0.4,0.003,0.04)$. The calculated AIC for the model with dynamic seasonal variation component was -169, whereas for the model with a static seasonal variation the AIC was −181, favoring the static model as being parsimonious.

**Figure 1:**
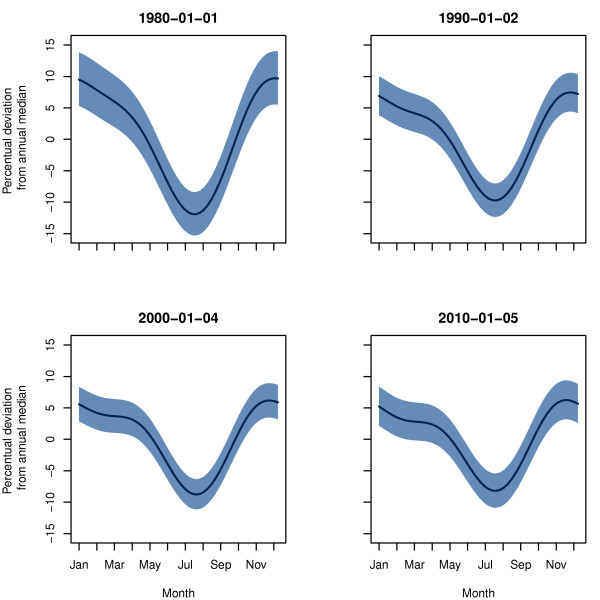
**Seasonal variation in incidence rates of stroke in AF patients.** The estimated seasonal variation in incidence rates of hospitalizations with stroke in AF patients in Denmark from 1980 to 2011 adjusted for an overall trend. The seasonal variation is presented as the percentual deviation from annual median for four time points.

**Figure 2:**
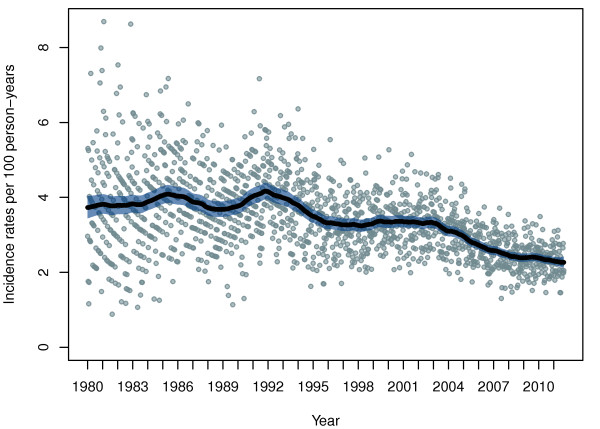
**Trend in incidence rates of stroke in AF patients.** The overall trend is presented as the underlying level of incidence rates of hospitalizations with stroke per 100 person-years for patients with AF in Denmark, adjusted for seasonal variations.

Figures 1 and 2 show the estimated seasonal variation and trend of incidence rates of stroke in AF patients. To illustrate the dynamic nature of the model, four instances of the seasonal variation are shown in Figure 1. The estimated seasonal variation from January to December is calculated according to the estimated parameters of the model for the chosen time points. In Figure 2, the estimated trend is shown, indicating a general decrease in incidence rates over time; however, with periods of local increase. The observed incidence rates are superimposed as bullets. Due to the nature of the open cohort design, where subjects may enter and leave the cohort at all times, the person-time at risk is relatively small compared with the numbers of hospitalizations at the beginning of the study period.

**Figure 3:**
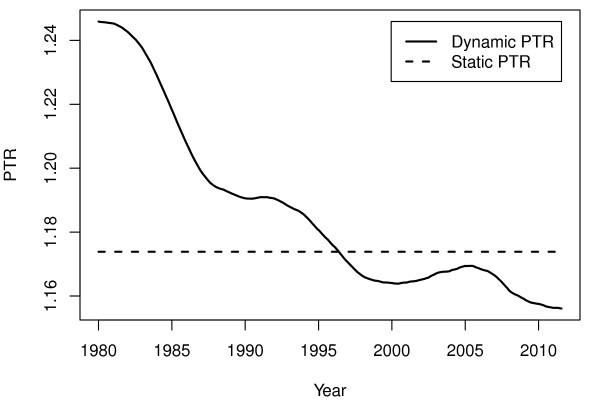
**Peak-to-trough ratios.** Peak-to-trough ratio of the seasonal variation in incidence rates of stroke in AF patients in Denmark estimated by a dynamic generalized linear model. The solid line represents the dynamic peak-to-trough ratio estimated by including a dynamic seasonal variation component in a generalized linear model, whereas the dashed line represents a static seasonal variation.

**Figure 4:**
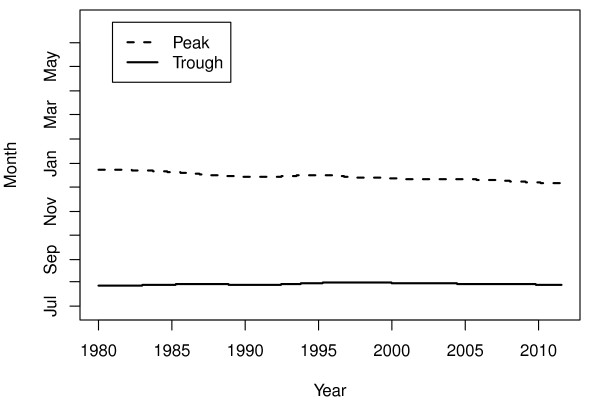
**Times for peaks and troughs.** Time of year for peak (dashed line) and trough (solid line) through the study period.

In Figure 3, the changing PTT ratios estimated each week are shown, represented by the solid line. This plot indicates that the PTT ratio has been decreasing since 1980, starting at 1.25 in January 1980 and ending at 1.16 in August 2011. The dashed line in Figure 3 represents the PTT ratio of the seasonal variation when modeled as being static. The time of year for trough was in August and September, and the time for peak was in December and January. The changes in time for peak and trough is shown in Figure 4, indicating time for peak drifting towards early December, whereas time for trough seems to remain in early August.

The sensitivity of the estimation algorithm in regard to starting values was assessed by providing different starting values to the algorithm and comparing the final estimates with the obtained estimates given above. In general, the normed distance from $\hat{\phi }$ to the final estimate obtained by an alternative starting value was small and the corresponding log likelihood values were close. The computation time of the EM algorithm increased as the normed distance between *ϕ*_0_ and the alternative starting value increased. However, the EM algorithm seemed to reach the same maximum for all alternative starting values, and accordingly, the computation time of the nlm routine did not change.

Based on investigation of residual plots in terms of autocorrelation plots and scatter plots of residuals versus fitted values, we identified no systematic deviations which would indicate misspecifications of the model.

## Discussion

This paper demonstrates an alternative statistical model to investigate seasonal variation of incidence rates of stroke in AF patients in Denmark, in terms of state space modeling, including a procedure to estimate hyperparameters, and propose a specific structure in specifying the linear predictor and covariance matrix between coefficients internally and over time. By adjusting for an overall trend, we were able to describe a changing seasonal variation during the study period from 1980 to 2011. The seasonal variation was modeled as a sum of two sinusoids with periods of 12 and 6 months, allowing the peak and trough to be non-symmetrically distributed during the calendar year. The characteristics of the seasonal variation in terms of the times for peak and trough changed only slightly during the study period, whereas the PTT ratio decreased substantially over time. The PTT ratio decreased from a 25% winter excess risk of being hospitalized with stroke having AF in 1980 to a 16% winter excess risk in 2011. Overall, the incidence rate of stroke in AF patients decreased from 4 per 100 person-years in January 1980 to 2 in August 2011, adjusted for seasonal variations.

The estimated overall trend indicated a decrease in incidence rates of stroke in AF patients during the study period. This result seems valid considering that during the late 1980’s and early 1990’s, several studies on AF and its consequences were published [35,36] and led to the introduction of oral anticoagulant treatment for patients with AF [37]. This treatment has a prophylactic effect on stroke in these patients, as also indicated by this study, where the overall trend in incidence rates of stroke in AF patients was decreasing since 1980. Furthermore, even more focus on patients with AF and the treatment of these patients has led to a constant decrease in the incidence rates of stroke in AF patients [38,39].

The seasonal variation estimated in the present study has the same overall characteristics as reported by other studies of seasonal variation in hospitalizations with stroke in AF patients in terms of time for peak in winter and trough in summer [40,41]. Such findings are reported for other cardiovascular diseases such as acute myocardial infarction, sudden death, and hypertension [42-45].

On the contrary, Boari et al., 2007 [46] found inconclusive results regarding seasonal variation of acute myocardial infarction in previous studies, reporting findings of peak during autumn or even summer, whereas others have not been able to identify any seasonal variation. Furthermore, inconclusive results have been found for other cardiovascular diseases [47]. These findings may be due to the amount and resolution of data and to the statistical model applied. Inappropriate statistical models for which one or several model assumptions are violated may provide invalid conclusions. One essential model assumption for Poisson regression is that the data is independent over time and not overdispersed. However, when investigating seasonal variation, data are expected to be internally dependent; hence, applying a Poisson regression model may not be appropriate. Real data assumed to be Poisson distributed may be overdispersed as well, therefore also violating the model assumptions for Poisson regression [12].

In the present study, we propose to apply a class of models which is a generalization of the GLMs, referred to as DGLMs. Due to the correlation structure of this model, both dependency between data and overdispersed Poisson distributed data may be modeled. These models generalize the GLMs and are naturally parameterized with meaningful parameters. When a modeler is used to apply GLMs, the conversion to DGLMs is rather straightforward, and interpretation of parameter estimates is familiar to the modeler. Also, when applying DGLMs in assessing seasonal variations in frequencies of hospitalisations, as opposed to GLMs, further details regarding model parameters may be provided. In Christensen et al., 2012, an analysis similar to the analysis proposed in this study and on the same data was performed [48]. A GLM, assuming data being Poisson distributed and specifying the linear predictor as a superposition of a seasonal variation with periods of 12, 6 and 3 months, and 6 weeks and an overall trend specified as a cubic spline, was fitted to the time series, *y*_*t*_, *t *= 1,…,*n*. This analysis provided a single set of parameter estimates and consequently a single estimate of the PTT ratio equal to 1.22. This figure may be considered as an average of the underlying possible evolvement of the true parameters. By applying a DGLM, this evolvement may be assessed.

When estimating the covariance structure of a DGLM, the modeler has to provide starting values for the estimation algorithm which may affect the estimated hyperparameters at convergence. We propose to apply the EM algorithm initially, to ensure convergence towards the region of the maximum rather fast, and switching to an iteratively numerical optimization algorithm to pinpoint the actual maximum. We investigated the robustness of the estimation scheme by applying different starting values. This investigation showed that for all given starting values, the final estimates were similar, indicating that the estimation procedure is rather robust in terms of starting values, results not shown.

Other models have been proposed in modeling seasonal variation, including generalized additive models, introduced by Hastie and Tibshirani, 1994, [12,17,49], and classic time series analysis [15,16,27,50,51]. As the objective is to describe the seasonal variation and changes herein over time exhibited by data, the DGLM is a useful approach since the correlation structure of the regression parameters is estimated explicitly, and is not considered being nuisance parameters as opposed to generalized estimation equations [15] and quasilikelihood approaches [16].

By fitting models with and without a dynamic seasonal variation component, we were able to assess whether the seasonal variation was in fact dynamic in terms of AIC. This criterion indicated that a static seasonal variation component was sufficiently describing the data and that the corresponding model was the most parsimonious model of the two models considered. However, when fitting a model with a dynamic seasonal variation, the present study revealed that the PTT ratio of hospitalizations with stroke in AF patients in Denmark changed from 1.25 to 1.16 during the study period. Considering this result from a clinical perspective, this change may still contain crucial information, since an incidence rate ratio reduction of 9 may seem worth investigating further.

## Conclusions

In conclusion, we propose to apply a DGLM when modeling seasonal variation in frequencies of hospitalizations with stroke in patients with AF. This model is capable of modeling serially correlated data and allows for parameters of the model to change gradually over time. The interpretation of the model is similar to that of ordinary Poisson regression, and when the modeler is familiar with ordinary regression analysis, the transition to DGLM is straightforward. Furthermore, the model may easily be adapted to other epidemiological contexts, when the objective is to study changes over time.

## Abbreviations

AF: Atrial fibrillation; AIC: Akaike’s Information Criterion; DGLM: Dynamic Generalized Linear Model; GLM: Generalized Linear Model; ICD: International Classification of Diseases; PTT: Peak-to-trough.

## Authors’ contributions

ALC made primary contributions to data collection and analysis, interpretation of results, and writing of the manuscript. SLC, KO, LHR, and CD contributed to the study conception and design. All authors contributed to interpretation of results, all revised the manuscript critically for important intellectual content, and all authors read and approved the final manuscript.

## Pre-publication history

The pre-publication history for this paper can be accessed here:

http://www.biomedcentral.com/1471-2288/12/174/prepub
